# Advantages of the use of ultrasound in newborn vascular access: a systematic review

**DOI:** 10.1007/s40477-023-00832-1

**Published:** 2023-10-06

**Authors:** Valentina Brusciano, Miriam Lecce

**Affiliations:** 1 Dipartimento di Scienze Biomediche Avanzate, Università degli Studi di Napoli Federico II, Naples, Italy; 2 Università degli Studi della Campania-Luigi Vanvitelli, Napoli, Italy

**Keywords:** Central venous access, Ultrasound, Newborns, Internal jugular vein, Subclavian vein, Femoral vein

## Abstract

Vascular access in neonates and small infants is often challenging. Ultrasound (US) screening and guidance improves its safety and efficacy. The advantages of a pre-implantation ultrasound examination are intuitive; it is a practical and safe technique that doesn’t use radiation, allowing static and dynamic evaluations to be carried out and identifying anatomical variations, the caliber and depth of the vessel, the patency of the entire course and attached structures (nerves, etc.). Optimization of the image is a crucial aspect in achieving a clear view of all anatomical structures while avoiding complications. The goal of this review was to look into the benefits of using US in invasive catheter insertion procedures, especially in pediatric patients. Ultrasonography is used to visualize vessels and related structures in two dimensions (2D), sometimes with the help of color Doppler to detect the presence of intraluminal thrombi by applying gentle compression to assess vessel collapse and evaluate morphologic changes in the internal jugular vein (IJV) who had undergone central venous catheter (CVC) insertion during the neonatal period (Montes-Tapia et al. in J Pediatr Surg 51:1700–1703, 2016).

## Introduction

The role of ultrasound in central vascular access placement is a critical issue. Recent literature describing the use of point of care ultrasound (POCUS) for various applications in the neonatal intensive care unit (NICU) has garnered increased interest among radiologists and neonatologists. Indeed obtaining vascular access in the neonate is a challenging and important aspect of their care [2, 3]. Multiple studies have demonstrated the endless advantages of using ultrasonography for vessel selection, such as the target vessel visualization to minimize difficulties related to anatomical variants. In fact anatomical variation may partly account for the inability to cannulate the internal jugular vein in certain patients. In these cases, ultrasound examination quickly establishes the position of the internal jugular vein and may allow for easy and rapid access. In particular, in neonatal patients the challenge is more complicated due to the small diameter of the vascular structures. Just consider that anatomical variants of the internal jugular vein that it is located anterolateral to the carotid artery in 92%,  > 1 cm lateral to the carotid artery in 1%, medially in 2%, and outside the pathway predicted by landmarks in 5.5% of patients [4]+fig n.1.

The placement of a CVC, despite being performed by experienced operators, is associated with 60–95% complications (depending on the entry site and type of patient) when performed using a blind technique; otherwise working knowledge of surface and deep anatomy minimizes complications [5].

Real-time ultrasound-guided US is safer and more efficient than a landmark approach.

If it is evident the efficacy of the US, it should be pointed) out safe use of US needs education and training to correctly interpret the images and achieve eye–hand coordination.

Moreover, vigilance remains necessary during insertion and maintenance and also following a recent failed insertion or the removal of a central venous catheter, to reduce any complications [6].

## Definition of vascular access and pre-procedural vein selection

The WoCoVA Foundation (WoCoVA = World Conference on Vascular Access) has developed an international Consensus to clarify the proper indication of central versus peripheral venous access; discuss the indications of the different peripheral venous access devices; to define the proper techniques of insertion and maintenance that should be recommended today [7, 8].

According to their latest guidelines venous access devices (VADs) are defined as peripheral or central based on the position of the tip of the catheter. Any VAD with the tip located in the superior vena cava (SVC) or in the inferior vena cava (IVC) or in the right atrium (RA) should be considered as a central venous access device (CVA).

More precisely, amoung central venous catheter (CVC), we define Centrally Inserted Central Catheter (CICCs) if the entry is at the level of the deep veins of the supraclavicular and subclavicular area, peripherally inserted central catheter (PICCs) as those with brachial insertion and femorally inserted central catheter (FICCs) as those with femoral insertion.

A peripheral VAD (PVAD) can be defined as any VAD with the tip not located in SVC or RA or IVC.

In pediatric patients and neonates, the choice of the brachiocephalic vein is increasingly in use, as it presents adequate size and favorable characteristics in its in-plane approach.

Given that the internal jugular and subclavian v. are generally smaller than the brachiocephalic trunk in any newborn, it is deductive which is the preferred choice for a catheter of at least 3 Fr in caliber.

Traditionally, CVC placement is performed using landmark techniques based on the knowledge of anatomic structures and palpation of arteries next to the veins. The use of surface anatomical findings is based on the presumed location of the vessels and blind puncture with needle insertion until blood is drawn into the syringe. The catheter is then inserted on a metal guide using a Seldinger technique [8]. These landmark techniques cannot account for anatomic variations at the CVC insertion site so the incidence of mechanical complications increases six-fold if more than 3 venipuncture attempts are made by the same operator. The use of ultrasound during the procedure can be used to easily visualize anatomic structures and confirm patency of the vein and thus help to avoid unintended arterial puncture or unsuccessful cannulation. In addition, US can facilitate CVC placement in special clinical situations in which landmark techniques based on palpation of the arterial pulse are challenging or impossible.

Although the ultrasound method has compared favourably with the landmark technique, its widespread use has been hampered by the impracticality of specially designed ultrasound devices or sterile scanner manipulation, unavailability of equipment, and lack of trained personnel.

## Technical aspects of tip navigation and tip location

As a first step, one should use US to identify the anatomy of the insertion site (vein and artery, adjacent anatomic structures) and the localization of the target vein. Then, The US allows confirmation that the needle tip is placed centrally in the vein before approaching the guide (tip location) and to verify its absence in the surrounding structures and to ascertain the central position of the tip (tip location) as recently described by Boris Brkljacic et al. [9].

It is clear that the use of ultrasound during the procedure significantly improves the success rate on the first attempt and reduces complications. Practical recommendations for the use of ultrasound during venous cannulation are reiterated by guidelines, associations, important consensus and government agencies such as the National Institute for Health and Clinical Excellence and the Agency for Healthcare Research and Quality’s evidence report [10, 11]. US can be used in two different ways: “Static” or “Indirect US” and “real-time or direct US”.

The former describes a technique using US only before CVC placement to identify the anatomy of the target vein and adjacent anatomic structures, including the patency of the vein and its dimensions and depth from the skin, the latter “real-time” US or “direct US” describes a technique of needle advancement and vessel puncture under permanent US control [12].

Ultrasound is most effective when used in real time (during needle progression) with sterile technique (sterile gel and probe covers). The needle is highlighted on the screen and simultaneously directed towards the target vessel away from the surrounding structures with an appropriate pathway [13].

**Fig. 1:**
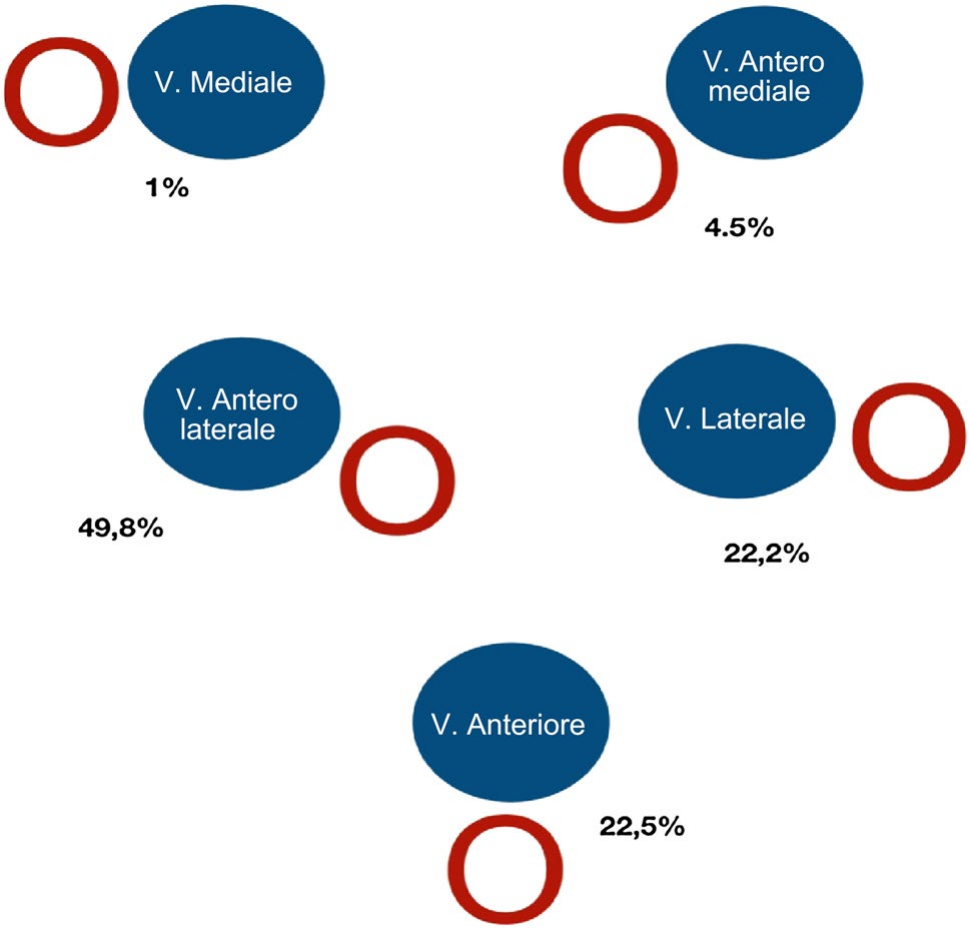
Anatomical variants of the internal jugular vein (IJV)

These techniques are however superior to the traditional blind approach with simple landmarks. A single operator can perform real-time ultrasound guided cannulation. The non-dominant hand holds the probe while the dominant hand handles the needle (Fig. 1).

The success of the procedure is confirmed by the direct view of the needle entering the vessel and the aspiration of blood into the syringe. The probe is placed on the sterile field and the metal guide on which the catheter or micro-introducer will be inserted, is introduced.

Elective venous access in NICU should always be performed in a sterile environment: an aseptic approach including covering the puncture site with a large sterile drape, using sterile barriers (hat, mask, sterile gloves, sterile body gown), and covering the ultrasound probe and cable with a sterile cover is shown. The position of the operator allows aligning the insertion site, the needle, and the ultrasound screen in the line of sight during needle insertion.

## Prevention and management of complications

Central venous catheter (CVC)-related complications can be life-threatening, with an estimated 12.5–25% mortality associated with catheter-related bloodstream infections (CRBSIs) [14].

Newborns are particularly vulnerable to complications and related symptoms can often be missed in these patients. Complications vary based on weight, age, comorbidity, and type of catheter access.

The most common complications of IJ vein cannulation are arterial puncture and hematoma [15]. The most common complication of SC vein cannulation is pneumothorax [16].

The incidence of mechanical complications increases sixfold when more than three attempts are made by the same operator.

**Fig. 2:**
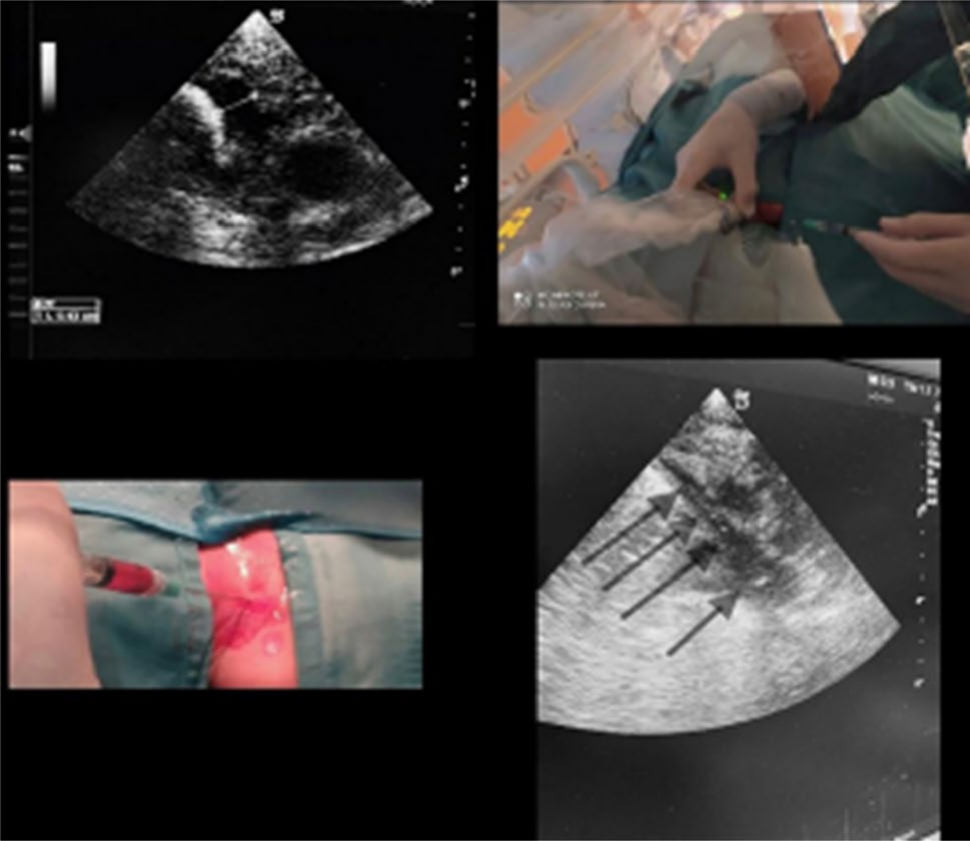
Guidewire was advanced through the needle in BCV while viewing the SVC from the supraclavicular fossa [24]

Many complications are caused by malpositioning of catheter and this situation is often identify by such fluoroscopy or chest X-ray in clinical routine. Although most clinicians use radiological imaging [17], it is not the gold standard technique, and also exposes the child to radiation [18, 19]. Ultrasound findings correlate well with chest radiography to confirm that the tip of the central venous catheter is positioned correctly. Using a 2−6 MHz convex or 2−5 MHz sector transducer, a second operator can obtain a real-time image through the modified subcostal 4-chamber view by gently rocking the probe until the entrance of the superior vena cava in the right atrium is observed. The tip of the central venous catheter is seen as a hyper echoic image; if necessary, advance or withdraw catheter so that it is positioned at the junction between the superior vena cava and the right atrium. Then fix the catheter to the skin with 3.0 monofilament sutures and covered with a sterile dressing [20]. At the end of the procedure, perform a final ultra-sound scan to rule out any possible complications, such as pneumothorax, pericardial effusion or tamponade and haemothorax. This will allow any major complications to be rapidly diagnosed and treated [20, 21] (Fig. 2).

The use of US to reduce the number of complications related to vascular access for CVC placement has been evaluated in numerous previous studies in a variety of clinical settings.

Recent Cochrane systematic reviews and meta-analyses summarize the current evidence for US guidance versus anatomic landmark techniques for CVC placement in the IJV [22, 23] with regard to complications of CVC placement. These meta-analysis included adult and pediatric patients treated in the intensive care unit or the operating room and compared conventional landmark techniques with techniques using static or real-time US or Doppler US.

## Conclusion

Real-time ultrasound guidance of CVC insertion provides the operator with visualisation of the desired vein and the surrounding anatomic structures prior to and during the insertion of the catheter. This method appears to improve the success rate and decrease the complication rate associated with CVC placement.

According to our literary analysis US guidance can improve patient safety and procedural quality during CVC placement in the IJV, FV, and SV.

In conclusion, it can be stated that the use of US is fundamental in venous cannulation procedures. The procedure is easy, does not require time and has multiple advantages which can be summarized in 7 step:

1. Identification of the anatomy of the entry site and location of the vein;
2. Examination of vessel patency;
3. Vein puncture with real time ultrasound technique;
4. Confirmation of needle position in the vein; 5. Tip navigation;
5. Tip location;
6. Ultrasonographic diagnosis of possible complications [23].

Recommended steps for ultrasound-guided placement of a central venous catheter in the brachiocephalic vein (BCV): check the patency of the vein; control the in-plane advance of the needle in real time; check the correct position of the wire guide; verify the position of the catheter inside the vein, and verify the correct location of the catheter tip at the junction of the superior vena cava (SVC) and right atrium (RA) [19].
